# Subclinical Ocular Changes after Intravitreal Injections of Different Anti-VEGF Agents for Neovascular Age-Related Macular Degeneration

**DOI:** 10.3390/jcm12237401

**Published:** 2023-11-29

**Authors:** Hisashi Matsubara, Ryunosuke Nagashima, Shinichiro Chujo, Yoshitsugu Matsui, Kumiko Kato, Manami Kuze, Mineo Kondo

**Affiliations:** 1 Department of Ophthalmology, Mie University Graduate School of Medicine, 2-174 Edobashi, Tsu City 514-8507, Mie, Japan; 2 Matsusaka Chuo General Hospital, 102 Kawaicho Konozomi, Matsusaka City 515-8566, Mie, Japan

**Keywords:** neovascular age-related macular degeneration, anti-vascular endothelial growth factor, VEGF, intraocular inflammation, aqueous flare, electroretinography, aflibercept, brolucizumab, faricimab

## Abstract

Intraocular inflammations (IOIs) have been reported to occur after intravitreal injections of brolucizumab, and one of their causes has been suggested to be drug-specific features. We evaluated the anterior chamber by the aqueous flare value (AFV) and the retina by flicker electroretinography (ERG) after the initial intravitreal injection of aflibercept (IVA), brolucizumab (IVBr), or faricimab (IVF) for neovascular age-related macular degeneration (nAMD). The AFV and flicker ERGs were determined before, 2 weeks after, and 4 weeks after the injections in 14 eyes of 14 patients for each drug. After the injections, none of the patients had an IOI, but the AFV increased significantly in the IVA and IVF groups. The increase in the IVF group was +4.6 photon count/ms, which was significantly greater than in the other groups, but was not clinically significant. The implicit time was significantly prolonged in the IVBr group but unchanged in the IVA and IVF groups. These results suggest that brolucizumab, administered at high molar doses, may cause transient retinal disturbances that are not detectable by general ophthalmologic examinations but affect the implicit ERG times.

## 1. Introduction

Intravitreal injections of anti-vascular endothelial growth factor (anti-VEGF) agents have been effective in improving the functional and anatomical properties of eyes with neovascular age-related macular degeneration (nAMD). At present, anti-VEGF treatments have become the first-line treatment for nAMD [1,2]. However, there are some shortcomings of anti-VEGF treatments, such as their weak suppression of exudations from the neovascular vessels and the need for frequent administration in some cases. To make the exudation control stronger and longer-lasting, new anti-VEGF agents have been developed and approved. Aflibercept (Eylea^®^, Regeneron Pharmaceuticals, Tarrytown, NY, USA) is a recombinant fusion protein of the human VEGFR-1 and VEGFR-2 fused to the fragment crystallizable region (Fc region) of the human immunoglobulin G1. It was approved in Japan in 2012 and is commonly used for nAMD. Brolucizumab (Beovu^®^ Novartis, East Hanover, NJ, USA) is a single-chain variable antibody fragment that was approved in 2020. Its low molecular weight allows higher molar doses to be administered compared with other commercially used anti-VEGF agents. Faricimab (Vabysmo^®^ Roche, Basel, Switzerland) is the newest anti-VEGF agent, which inhibits VEGF-A and angiopoietin-2, and it was approved for use in Japan in 2022. Clinical trials using these agents have demonstrated their efficacy in improving the visual acuity and anatomical structure of the retina [3,4,5]. However, unexpected intraocular inflammation (IOI) and retinal vascular occlusion (RO) were reported as adverse ocular complications in the clinical studies of brolucizumab [6,7,8]. Since those reports, there has been a growing interest in determining the cause of the development of IOIs after intravitreal injections of anti-VEGF agents.

IOI and RO are complications that are not specific to brolucizumab treatment, and anterior-chamber inflammation has also been reported after intravitreal aflibercept treatments (19%), while much lower rates were reported after ranibizumab treatments (2%) [9]. The incidence of IOI and RO was higher in patients treated with brolucizumab than with aflibercept [6,9]. The exact causes of the IOI and RO after anti-VEGF drug injections have not been determined, but it has been suggested that the properties of the individual anti-VEGF agent may be related to the development of the IOI and RO [10]. Thus, the different agents may produce different agent-specific subclinical responses after their injections.

Generally, ocular inflammatory responses after intravitreal injections can be confirmed by general ophthalmic examinations, e.g., slit-lamp examinations, ophthalmoscopy, and optical coherence tomography (OCT). Laser flare-cell photometry can also be used to determine the amount of proteins in the aqueous humor, and the values are designated as the aqueous flare values (AFVs). A positive finding would indicate a breakdown of the blood–ocular barrier. There have been reports of changes in the AFVs in IOI cases after brolucizumab treatment that were detected by slit-lamp and fundus examinations [7,8]. However, laser flare photometry can detect milder changes in the AFVs, indicating blood–ocular barrier changes more accurately than slit-lamp or fundus examinations. Therefore, laser flare-cell photometry may detect an inflammation after intravitreal injection that general ophthalmic examinations cannot detect, i.e., subclinical changes. Although there have been reports of increases in the AFV after aflibercept or ranibizumab injections for nAMD [11,12], there have been no reports of subclinical changes in the AFV after treatments with brolucizumab and faricimab without IOI and RO. Therefore, whether there are AFV changes after treatment with brolucizumab and faricimab has not been definitively determined.

Traditionally, electroretinography (ERG) has been used as an objective method to detect alterations in the retinal physiology, and because of its sensitivity it can detect mild-to-subclinical retinal alterations. However, conventional ERG testing, i.e., the use of a contact lens electrode to pick up the electrical signals, is somewhat invasive and time-consuming. Fortunately, a portable ERG device that uses skin electrodes to pick up the electrical signals has recently been introduced. This is especially helpful in patients who have undergone intraocular surgical procedures [13,14]. However, it has not been generally used to determine the effects of anti-VEGF injections on the eye.

Thus, the purpose of this study was to determine whether there are subclinical changes in the blood–aqueous barrier and the physiology of the retina after anti-VEGF treatments with different anti-VEGF agents. To accomplish this, we recorded the AFV and flicker ERGs before and after an initial intravitreal injection of aflibercept (IVA), brolucizumab (IVBr), or faricimab (IVF).

## 2. Materials and Methods

### 2.1. Study Design and Ethics

The procedures used in this study were approved by the Ethics Committee of Mie University Hospital (approval #H2021-088), and they conformed to the tenets of the Declaration of Helsinki. This was a retrospective study, and the medical records of nAMD patients treated with IVA, IVBr, or IVF at Mie University Hospital, Mie, Japan between 1 September 2020 and 31 March 2023 were analyzed.

### 2.2. Inclusion and Exclusion Criteria

Patients were included if they were >50 years of age, clinically diagnosed with nAMD, and had begun anti-VEGF treatment with aflibercept, brolucizumab, or faricimab or had switched their anti-VEGF treatment to these agents. All of the eyes had recordings of both the AFV and flicker ERGs before and 2 and 4 weeks after the initial injection of each type of anti-VEGF agent. Patients were excluded if they had received any of the following treatments within 6 months prior to baseline: intraocular surgery, photodynamic therapy, laser photocoagulation, posterior capsulotomy, and intravitreal or subtenon injections of steroids. Patients were also excluded if they had prior vitrectomy, topical steroids, or uveitis of the studied eye.

### 2.3. Data Acquisition

Data were obtained before the first injection of each drug (baseline), two weeks after the initial injection (week 2), and four weeks after the injection (week 4). The demographic and clinical characteristics were extracted from the medical records, including the age, sex, number of anti-VEGF injections, interval between the last injections (weeks), decimal best-corrected visual acuity (BCVA), central subfield foveal thickness (CSFT), residual subretinal fluid (SRF), residual intraretinal fluid (IRF), intraocular pressure (IOP), AFV, and amplitudes and implicit times of the flicker ERGs. The changes in the AFV (ΔAFV), amplitude (Δamplitude), and implicit time (Δimplicit time) from the baseline to week 2 were calculated.

#### 2.3.1. Central Subfield Foveal Thickness (CSFT), SRF Detection, and IRF Detection

The CSFT was measured as the average thickness in the central 1 mm of the Early Treatment Diabetic Retinopathy Study 9-zone grid. It was measured as the distance between the internal limiting membrane and Bruch’s membrane in the OCT images obtained by a spectral-domain OCT (Spectralis HRA2; Heidelberg Engineering, Heidelberg, Germany) device. A 30° × 25° volume scan of the macula was performed at the baseline to identify areas of macular neovascularization (MNV), SRF, and IRF. Then, at week 2 and week 4, a volume scan was performed including the area where the SRF and IRF were present at the baseline to evaluate the presence or absence of SRF and IRF.

#### 2.3.2. Measurements of Aqueous Flare Value (AFV)

The AFV was measured by laser flare-cell photometry (Kowa FC-1000 LFCM; Kowa Co, Ltd., Nagoya, Japan) at least 30 min after the instillation of topical 0.5% tropicamide and 0.5% phenylephrine (Mydrin-P, Santen Co., Osaka, Japan). The mean of five reliable measurements was used for the statistical analyses. The mean value was expressed in photon counts/ms (pc/ms).

#### 2.3.3. Flicker ERG Recordings with the RETeval^TM^ ERG System

Full-field flicker ERGs were recorded with the RETeval^TM^ (Mayo, Inazawa, Japan) ERG system. The ERGs were picked up by a skin electrode array (Sensor Strip; LKC Technologies, Inc., Gaithersburg, MD, USA) that was placed 2 mm from the margin of the lower eyelid. Before the ERG recordings, the pupils were fully dilated with topical 0.5% tropicamide and 0.5% phenylephrine (Mydrin-P, Santen Co., Osaka, Japan). Full-field white stimuli were presented with a 60 mm diameter Ganzfeld dome, and the white stimuli were created by a combination of 3 colored light-emitting diodes. The luminance of the stimuli was 3 cd·s/m^2^ flash with a 30 cd/m^2^ background light (ISCEV standard), and the frequency of flicker stimulation was 28.3 Hz. The amplitudes and implicit times of the fundamental component were automatically measured and displayed by the RETeval^TM^ system using a special algorithm incorporating discrete Fourier transformation and cross-correlation analysis. We analyzed the amplitudes and implicit times of the fundamental components of the flicker ERGs, which were sensitive to functional changes in the retina [12,13,14,15]. The amplitudes and implicit times were expressed in microvolts (μV) and milliseconds (msec), respectively.

### 2.4. Statistical Analyses

The descriptive data are presented as numbers and medians with the 25th and 75th percentiles (Q1 and Q3, respectively). For the statistical analyses, chi-squared tests, Mann–Whitney U tests, Wilcoxon signed-rank tests, Kruskal–Wallis tests with post hoc Dunn’s tests, and Friedman tests with post hoc Dunn’s tests were used. The decimal BCVA was converted to the logarithm of the minimum angle of resolution (logMAR) units for the statistical analyses. All *p*-values were two-sided, and *p* < 0.05 was taken to be statistically significant. All statistical analyses were performed using Prism software (version 9.0) (GraphPad, Inc., La Jolla, CA, USA).

## 3. Results

### 3.1. Demographics of Patients

The demographics of all of the patients are shown in Table 1, with the patients separated by the anti-VEGF agent used. Forty-seven eyes of forty-seven patients were studied. Five eyes were excluded because the implicit times or amplitudes of the ERGs could not be measured. In the end, 42 eyes of 42 patients were analyzed, and there were 30 men and 12 women. Each of the IVA, IVBr, and IVF groups had 14 eyes. Thirteen eyes in the IVA group were treatment-naïve and one eye was a recurrence case after a long period of inactive MNV. In the IVBr group, 13 of the 14 eyes were switched from aflibercept and 1 eye was switched from faricimab to brolucizumab. In the IVF group, 1 eye was treatment-naïve and 13 eyes were switched from aflibercept to faricimab. Drugs were switched at the discretion of the treating physician, and they were switched when the SRF and IRF were not resolved with IVA at intervals of ≤8 weeks, when the SRF and IRF were resolved with IVA at intervals of ≤7 weeks, or when IVF did not control the SRF or IRF. The drug that they switched to was determined by the treating physician. The IVA group was significantly younger than the other groups. All eyes in the IVA group were phakic, seven eyes were phakic in the IVBr group, and nine eyes were phakic in the IVF group. According to the self-reports, the number of patients with diabetes mellitus was zero in the IVA group, two in the IVBr group, and one in the IVF group. The differences in the last injection interval and the number of prior injections between the IVBr and IVF groups were not significant. The AFV in the IVA group was significantly lower than that in the IVBr group (*p* = 0.047 by Dunn’s test), but it was not significantly different from that of the IVF group (*p* = 0.30, by Dunn’s test). The amplitudes and implicit times of the flicker ERGs at the baseline were not significantly different among the three groups.

### 3.2. Effectiveness of Anti-VEGF Treatment on Best-Corrected Visual Acuity (BCVA), Intraocular Pressure (IOP), Central Subfield Foveal Thickness (CSFT), Residual Subretinal Fluid (SRF), and Residual Intraretinal Fluid (IRF) 

The changes in the BCVA, IOP, CSFT, SRF, and IRF are shown in Table 2 according to the anti-VEGF agents used. No ophthalmic or systemic complications were observed in any of the cases after each type of anti-VEGF injection. Slit-lamp and fundus examinations showed no IOI or RO associated with the intravitreal injections in any of the cases. The BCVA improved significantly in the IVA group and was maintained in the IVBr and IVF groups. The IOP did not increase significantly in any of the groups. The CSFT in all groups decreased significantly from that at the baseline by week 2 and week 4. The number of cases with SRF or IRF did not increase from that at the baseline by weeks 2 and 4.

### 3.3. Aqueous Flare Values (AFVs)

**Figure 1:**
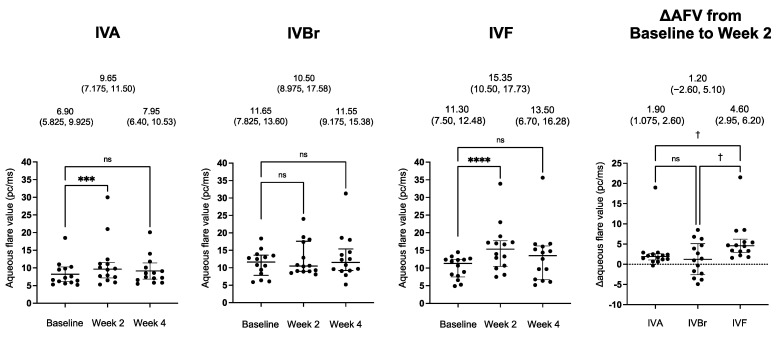
Plot of the aqueous flare values (AFVs) and the changes in the AFVs from the baseline to week 2 (ΔAFV) in the IVA, IVBr, and IVF groups. The median and error bars represent the 25th and 75th percentiles. Each point is one patient. Data at the top are the medians (the 25th and 75th percentiles). The AFVs were significantly increased at week 2 in the IVA and IVF groups, and then decreased at week 4. The AFV did not significantly increase at either week 2 or week 4 in the IVBr group. The ΔAFV in the IVF group was significantly greater than that in the IVA and IVBr groups; *** *p* < 0.001, **** *p* < 0.0001, Friedman test with a post hoc Dunn’s test; ^†^ *p* < 0.05, Kruskal–Wallis test with a post hoc Dunn’s test, ns (not significant).

The AFVs and the ΔAFVs after injection in all groups are shown in Figure 1. The median AFV of the IVA group was less than 10 pc/ms at all examination times. The AFVs were significantly increased at week 2 in the IVA group (*p* = 0.0007; Dunn’s test) and the IVF group (*p* < 0.00001; Dunn’s test), while they were decreased at week 4. On the other hand, the AFVs did not increase significantly at either week 2 or week 4 in the IVBr group (*p* = 0.81, Friedman test). The ΔAFV in the IVF group was significantly greater than that in the IVA and IVBr groups (IVA vs. IVF, *p* = 0.029; IVBr vs. IVF, *p* = 0.022; IVA vs. IVBr, *p* > 0.99, by the Kruskal–Wallis test and Dunn’s test). The median ΔAFV was 1.90 pc/ms for the IVA group, 1.20 pc/ms for the IVBr group, and 4.60 pc/ms for the IVF group.

### 3.4. Electroretinograms (ERGs)

#### 3.4.1. Amplitudes 

**Figure 2:**
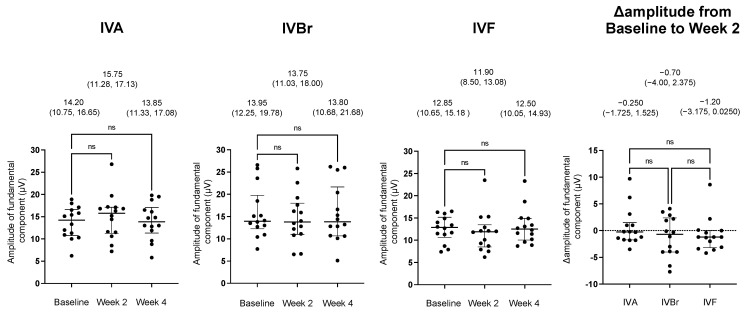
Plot of the amplitudes of the flicker ERGs and the changes in amplitude from the baseline to week 2 (Δamplitude) in the IVA, IVBr, and IVF groups. The medians and error bars represent the 25th and 75th percentiles. Each point is one patient. Data at the top are the medians (the 25th and 75th percentiles). There was no increase at either week 2 or week 4 in any of the groups. There were no significant differences in the Δamplitudes among the three groups. We used the Friedman test with a post hoc Dunn’s test, ns (not significant).

The amplitudes and the Δamplitudes of the flicker ERGs after the injections are shown in Figure 2 for all groups. There was no significant increase in the Δamplitudes at either week 2 or week 4 in any og the groups. There were no significant differences in the Δamplitude among the three groups.

#### 3.4.2. Implicit Times

**Figure 3:**
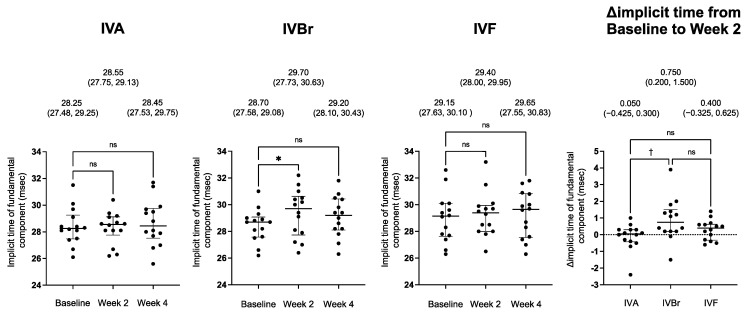
Plot of the implicit times after the injection and the changes in the implicit times from the baseline to week 2 (Δimplicit time) in the IVA, IVBr, and IVF groups. The medians and error bars represent the 25th and 75th percentiles. Each point is one patient. Data at the top are the medians (the 25th and 75th percentiles). There was no prolongation at either week 2 or week 4 in the IVA and IVF groups. In the IVBr group, the implicit time was significantly prolonged at week 2 but reduced to the baseline at week 4. The Δimplicit time in the IVBr group was statistically more significant than that in the IVA group; * *p* < 0.05, Friedman test with a post hoc Dunn’s test; ^†^ *p* < 0.05, Kruskal–Wallis test with a post hoc Dunn’s test, ns (not significant).

The implicit times and the Δimplicit times after the anti-VEGF injections are shown in Figure 3 for all groups. The implicit time in the IVBr group was significantly prolonged from the baseline at week 2 (*p* = 0.016, by Dunn’s test), but not at week 4. There was no significant prolongation at either week 2 or week 4 in the IVA and IVF groups. The number of cases with prolongation of more than 1.0 msec at week 2 was one in the IVA group, seven in the IVBr group, and two in the IVF group. The Δimplicit time in the IVBr group was significantly greater than that in the IVA group (*p* = 0.021, by Dunn’s test).

## 4. Discussion

The findings showed that the AFVs of the IVA and IVF groups were significantly increased at week 2 and then recovered to the baseline values at week 4. However, in the IVBr group, the AFV was not increased significantly at week 2 or week 4. The ΔAFV of the IVF group was significantly greater than that of the other groups, but the increase was slight and not significant. The implicit times of the flicker ERGs were significantly prolonged at week 2 and then were not significantly longer than the baseline at week 4 in the IVBr group. The Δimplicit time of the IVBr group was significantly longer than that of the IVA group. On the other hand, the amplitudes did not change significantly in any of the groups. These results indicate that faricimab affected the AFV while brolucizumab affected the implicit times of the flicker ERGs.

The AFV represents the protein concentration in the aqueous humor. Thus, an increase in the AFV indicates a disruption of the blood–aqueous barrier, leading to a leakage of proteins into the anterior chamber. In nAMD eyes, the AFV is higher than that of normal eyes [15] and is lower in eyes with milder lesion activity that does not require anti-VEGF medications [12]. During the bevacizumab treatments for nAMD, higher AFVs have been reported in older patients, pseudophakic patients, and patients with residual IRF [16]. The patients in the IVA group were significantly younger than those in the IVBr and IVF groups. All cases in the IVA group were phakic, but 7 of 14 in the IVBr group and 5 of 14 in the IVF group were pseudophakic. Eight of the forty-two patients had IRF at the baseline, but the number did not differ significantly among the three groups. In addition, cases in the IVBr and IVF groups had longer treatment durations and more frequent intravitreal injections. In eyes with AMD, the AFV has been reported to increase as the course of treatment lengthens and the exudation persists [16]. The persistent retinal exudation and the trauma caused by the frequent vitreous injections may have also induced the inflammation. These findings suggest that differences in age, lens status, treatment duration, history of frequent injections, and persistent exudation may have contributed to the differences in AFV at the baseline in the three groups.

The degree of change in the AFV after the anti-VEGF injections was different for the different anti-VEGF agents. In earlier studies examining the differences in the AVF between eyes treated with bevacizumab and ranibizumab one day after the injection, the AFVs were greater after the bevacizumab injection but not after the ranibizumab injection. However, the increase was 2.7 pc/ms, and it was concluded that the increase in the AFV might not be clinically significant [11]. In our study, the median change was 1.9 pc/ms for the IVA group, 1.2 pc/ms for the IVBr group, and 4.6 pc/ms for the IVF group. All of these changes were not significantly different from those reported previously. There are differences in the molecular structure of the three anti-VEGF agents. The Fc region of the human immunoglobulin G1 activates complement-mediated cytotoxicity and the Fc receptors on immune cells. Therefore, the structural difference of the Fc region induces an inflammatory reaction triggered by the Fc antibody region. Aflibercept is a recombinant fusion protein that has the Fc region of the human immunoglobulin G1 [17,18]. Brolucizumab is a single-chain variable fragment molecule that targets VEGF-A and lacks the Fc region [19]. Faricimab is a bispecific antibody, and its FcRn- and FcγR-binding sites are modified to disable the Fc-mediated effector functions of the antibody [20]. Therefore, faricimab was expected to induce weaker inflammatory responses than aflibercept because of its modified Fc domain. However, our results showed that the AFV of the IVF group was higher than that of the IVA group in spite of the structural specificity of the FcγR-binding site. The ΔAFV of both the IVA and IVF groups was not as large as that in the presence of an inflammatory response. Therefore, it is not likely that the induction of inflammation by anti-VEGF drug injections was responsible for the slight increase in the AFVs.

Factors other than inflammation probably contributed to the increase in the AFV. Laser photometric measurements of flares and cells in the anterior chamber can be affected by some non-disease factors that alter the aqueous humor protein levels [21]. In addition to inflammation, the effects of the concentration of the protein and the molecular mass of the protein in solution should also be considered as causes of the increased AFV after the intravitreal anti-VEGF agent injections. Bovine serum albumin (BSA) and human immunoglobulin G (IgG) have been used as proteins in previous studies of laser flare-cell photometry [22,23]. The molecular mass of BSA is 66 kDa, that of IgG is 150 kDa, that of aflibercept is 97 to 115 kDa, and that of faricimab is 149 kDa. Thus, aflibercept and faricimab should be detectable by laser flare-cell photometry. Once a drug is injected into the vitreous body, it slowly diffuses into the anterior chamber and can be detected in the aqueous humor. However, to the best of our knowledge, there have been no studies that examined whether the AFV is altered by anti-VEGF drugs entering the anterior chamber. After a single intravitreal injection of 6.0 mg of faricimab in nAMD eyes, the concentration of faricimab in the aqueous humor was measured to be 92.5 μg/mL seven days later [24]. Its half-life in the aqueous humor was 7.61 days [24]. Therefore, the concentration of faricimab was about 46 μg/mL at 14 days after the injection. A linear regression equation for the photon count and IgG concentration in an in vitro study has been reported as follows [23]:

log (photon count) (pc/ms) = 1.23 log (IgG concentration) (mg/dL) − 0.17.

The increase in the AFV should be 4.4 pc/ms in the aqueous humor 14 days after a faricimab injection, according to this formula. This value did not differ significantly from the increase in the AFV found in the IVF group. This suggests that the faricimab molecules that diffused into the anterior chamber may have been the cause of the increase in the AFV. Two studies have reported that the larger the molecular mass of the protein, the greater the AFV will be [22,23]. In addition, the half-life of the different drugs is different, and the half-life also affects the AFV. Aflibercept has a smaller molecular mass and a shorter half-life than faricimab. It was detected for approximately 115 h in the vitreous body of rabbit eyes after an intravitreal injection [25]. Thus, these differences may have led to a lesser increase than faricimab.

In contrast, it has not been determined whether brolucizumab can be detected by laser-flare cell photometry. The molecular mass of brolucizumab is 29 kDa, which is the smallest of the three agents tested, and the half-life of brolucizumab in the aqueous humor of rabbit eyes was 65.2 h [26]. Based on these findings, the residual amount of the drug two weeks after administration was calculated to be 2.8% in the aqueous humor for brolucizumab. Even after considering the differences between animal and human experiments and the different measurement sites, brolucizumab is unlikely to remain in the aqueous humor longer than aflibercept or faricimab. Thus, the differences in the AFVs among these drugs may be caused by the differences in their detectability by laser flare-cell photometry due to differences in the molecular mass and the amount of residual drug in the eye, due to differences in the half-lives of the drugs. Therefore, the increase in the AFV of aflibercept and faricimab was considered not to be clinically significant and should not cause aseptic inflammations.

The flicker ERGs originate from the cone system including the ON–OFF bipolar cells, and it is known that different intraocular changes can affect the implicit times and amplitudes of the flicker ERGs. Thus, the implicit times can be prolonged in eyes with mild retinal abnormalities [27]. Our results showed that the amplitudes did not change after the anti-VEGF injections in any of the three groups, and the Δamplitude did not differ between groups. These findings are similar to those of an earlier report on the effects of anti-VEGF treatment on the flicker ERGs [14]. The lack of significance may have been due to the large individual variations in the amplitudes of the flicker ERGs and the small sample size. On the other hand, the implicit times were significantly prolonged, but only in the IVBr group. The possible explanations for this include increases in the IOP, exudations, and inflammations, along with the suppression of VEGF. An earlier study reported elevated IOP immediately after an anti-VEGF injection, which affected the ERGs [28]. However, our results showed no significant increases in the IOP at week 2 and week 4, indicating that the changes in the flicker ERGs were not due to an elevation of the IOP. The number of cases with SRF or IRF did not increase, and the CSFT decreased significantly, indicating that the exudations associated with the nAMD did not affect the ERG parameters. Because the anti-VEGF drug injection is directly into the vitreous body and is the cause of the inflammation, it is likely that the inflammation will occur initially in the posterior chamber and spread to the anterior chamber, resulting in the increased AFV. Therefore, the results showing that the IVA and IVF groups had increased AFVs without prolongation of the implicit times also supported the idea that the increase in AFV in the IVA and IVF groups was unlikely to have been inflammation-induced.

It is known that intravitreal injections of anti-VEGF drugs will affect the retina and, thus, the implicit times and amplitudes of the flicker ERGs. Some animal studies have found that the implicit times and amplitudes of the ERGs were affected after the intravitreal injections of anti-VEGF agents [29,30]. In addition, it has been reported that the implicit times of the ERGs were prolonged in eyes after intravitreal injections of anti-VEGF agents and in the contralateral eyes 24 hours after the injection [14]. These results suggest that the pharmacological properties of the anti-VEGF drugs may prolong the implicit times. 

It is also known that anti-VEGF drugs affect the ocular circulation. It has been reported that the ocular perfusion [31,32,33] and the choroidal thickness decreased after IVA or IVBr injections [34,35]. A laser blood flowmeter was used to show that ranibizumab decreased the retinal arterial vessel diameter, velocity, and blood flow in patients with nAMD who had received fewer ranibizumab treatments [36]. Laser speckle flowgraphy showed a decrease in the retinal arterial and choroidal perfusion at 9 and 35 days after a single anti-VEGF treatment in treatment-naïve nAMD patients [37]. Twenty-three cases of cotton wool spots were reported following anti-VEGF treatment for cystoid macular edema (CME) associated with CRVO, and the results suggested an association with acute transient retinal ischemia induced by the anti-VEGF agents [38]. The results of these studies indicate that exposure to anti-VEGF agents can decrease the retinal blood flow. The exact mechanism causing the reduction in the retinal blood flow after anti-VEGF drug injections has not been definitively determined. However, it has been suggested that this may be due to a decrease in the nitric oxide (NO) levels caused by the VEGF suppression [39,40].

The implicit times of the flicker ERGs are known to be prolonged by a reduction in retinal perfusion. It has been reported that the implicit times of the ERGs were slightly prolonged in patients with background diabetic retinopathy with minor ischemia [41]. The implicit times of the flicker ERGs were prolonged and the amplitudes were decreased in eyes with a central retinal-vein occlusion, which is a more intense ischemia of the retina [42,43,44]. Brolucizumab has a smaller molecular mass than the other anti-VEGF drugs and can be used at a concentration of ≥120 mg/mL. Thus, the anti-VEGF binding capacity of brolucizumab is 11 times greater than that of aflibercept and 5.8 times greater than that of faricimab [19]. This property could have prolonged the inhibition of VEGF activity and may have affected the retinal circulation after the intravitreal injection. In fact, in a report evaluating the ocular perfusion at 30 min after IVA or IVBr injections in nAMD eyes, there were 3 of 10 patients in the IVBr group with perfusion reductions of 30% or greater, compared to 0 patients in the IVA group [33]. Together with the high complication rates of RO after IVBr, these results indicate that the reduction in ocular perfusion with brolucizumab may be different from that caused by other anti-VEGF drugs. In a previous report, the mean prolongation of the implicit times of the flicker ERGs at 24 h after the anti-VEGF treatment was 1.0 msec in the treated eyes and 0.8 msec in the untreated eyes [14]. In our cohort, the number of cases with prolonged implicit times of >1.0 msec was greater in the IVBr group than in the other two groups. In the IVBr group, the implicit times were significantly prolonged at week 2, and the changes in the implicit times were significantly greater than those in the IVA group. Furthermore, the implicit times were subsequently reduced and did not differ from the baseline values. The amplitudes of the flicker ERGs did not change significantly after IVBr. These results suggest that the implicit times may have been prolonged by the anti-VEGF effects of brolucizumab on the retinal blood flow, and that the changes in the retinal circulation might be transient and not large enough to cause a permanent prolongation of the implicit times.

There were major limitations of this study, including its retrospective design, the small number of cases for each anti-VEGF group, and the lack of a control group such as the contralateral eye. There was also a mixture of treatment-naïve and previously treated cases. Because the therapy for nAMD is initiated with aflibercept in most cases in Japan, the number of patients who switched from other anti-VEGF agents to aflibercept was small. As a result, almost all aflibercept-initiated patients were treatment-naïve cases. In addition, because of the risk of IOI after IVBr and the shortness of the interval since the approval of faricimab, almost all of the subjects in the IVBr and IVF groups had previously been treated with aflibercept and switched to one of the other drugs. Although the evaluations were performed two weeks after the injection, it may have been more meaningful to have evaluated the effects of the anti-VEGF drugs after one day or one week. Furthermore, other limitations regarding the flicker ERGs included the lack of evaluation of the time to normalization of the IOP after the injection, the lack of measurements of the axial lengths, and the inability to exclude the effects of changes in retinal morphology due to the anti-VEGF treatment. Another limitation was that only the flicker ERGs were recorded, and ERGs under scotopic conditions were not examined. Because there are more rod than cone photoreceptor cells in the retina, the scotopic ERGs may be more sensitive to the intraocular changes and represent the retinal state more accurately. Thus, further studies, such as recording the scotopic responses after the administration of anti-VEGF drugs, could be informative.

## 5. Conclusions

The results show that the AFV increased after injections with IVA and IVF, which are high-molecular-mass formulations, but the increase was slight. These increases were most likely not the result of the inflammation induced by intravitreal injection of these drugs, and they were not considered to be clinically significant. After the intravitreal injection of brolucizumab, which has a low molecular mass and can be administered in higher molar doses, the implicit times of the flicker ERGs were transiently prolonged. This may have been due to an alteration of the retinal circulation by the potent VEGF suppression of brolucizumab. Both the AFV and implicit time changes are subclinical reactions that are undetectable by general ophthalmologic examinations. Further research is required to assess the changes in AFV and ERG after the administration of anti-VEGF agents for nAMD.

## Figures and Tables

**Table 1:** Patient characteristics.

	IVA Group	IVBr Group	IVF Group	*p*-Value
Sex (men, women) ^(1)^	8:6	11:3	11:3	0.35
Age (median (Q1, Q3)) ^(3)^	68.5 (62.0, 74.25)	77.5 (72.5, 80.0)	78.5 (71.5, 87.0)	0.0015 **
Number of injections (median (Q1, Q3)) ^(2)^	None	22.5 (10.0, 34.5)	35.5 (22.75, 56.25)	0.067
Last interval of injection (weeks) (median (Q1, Q3)) ^(2)^	None	4.5 (4.0, 6.5)	6.0 (4.0, 7.25)	0.38
BCVA (median (Q1, Q3)) ^(3)^	0.097 (0.0345, 0.325)	0.16 (0.0841, 0.265)	0.046 (−0.0198, 0.6990)	0.25
IOP (mmHg) (median (Q1, Q3)) ^(3)^	12.85 (11.0, 14.78)	12.15 (10.93, 16.30)	11.85 (10.50, 18.0)	0.66
CSFT (μm) (median (Q1, Q3)) ^(3)^	441.5 (344.8, 495.3)	373.0 (302.0, 608.0)	328.5 (237.3, 467.3)	0.28
Lens status (phakia:pseudophakia) ^(1)^	14:0	7:7	9:5	0.011 *
SRF (Yes:No) ^(1)^	10:4	13:1	10:4	0.28
IRF (Yes:No) ^(1)^	3:11	2:12	3:11	0.86
AFV (pc/ms) (median (Q1, Q3)) ^(3)^	6.9 (5.825, 9.925)	11.65 (7.825, 13.60)	11.30 (7.5, 12.48)	0.047 *
Amplitude (μV) (median (Q1, Q3)) ^(3)^	14.20 (10.75, 16.65)	13.95 (12.25, 19.78)	12.85 (10.65, 15.18)	0.40
Implicit time (msec) (median (Q1, Q3)) ^(3)^	28.25 (27.48, 29.25)	28.70 (27.58, 29.08)	29.15 (27.63, 30.10)	0.56

BCVA: best-corrected visual acuity, IOP: intraocular pressure, CSFT: central subfield foveal thickness, SRF: subretinal fluid, IRF: intraretinal fluid, ^(1)^ chi-squared test, ^(2)^ Mann–Whitney U test, ^(3)^ Kruskal–Wallis test with a post hoc Dunn’s test; * *p* < 0.05, ** *p* < 0.01.

**Table 2:** Changes in BCVA, IOP, CSFT, SRF, and IRF after injection in the IVA, IVBr, and IVF groups.

	Baseline	Week 2	Week 4	*p*-Value
**BCVA (logMAR)**				
IVA ^(1)^	0.097 (0.0345, 0.325)	-	0.046 (−0.01975, 0.195)	0.012 *
IVBr ^(1)^	0.16 (0.08425, 0.265)	-	0.19 (0.097, 0.24)	0.69
IVF ^(1)^	0.046 (−0.01975, 0.30)	-	0.0715 (0, 0.325)	0.82
**IOP (mmHg)**				
IVA ^(2)^	12.85 (11.0, 14.78)	12.65 (11.00, 14.33)	12.35 (9.925, 14.18)	0.31
IVBr ^(2)^	12.15 (10.93, 14.08)	11.00 (10.53, 12.48)	11.0 (10.0, 12.35)	0.024 *
IVF ^(2)^	11.85 (10.50, 13.90)	11.50 (10.53, 13.43)	11.70 (10.23, 13.25)	0.3
**CSFT (μm)**				
IVA ^(2)^	441.5 (344.8, 495.3)	331.5 (286.8, 359.3)	330.0 (285.3, 352.5)	0.011 *
IVBr ^(2)^	373.0 (302.0, 608.0)	299.5 (264.5, 482.8)	314.0 (261.8, 506.8)	0.00040 *****
IVF ^(2)^	328.5 (237.3, 467.3)	307.0 (240.5, 377.0)	318.5 (234.5, 347.5)	0.0042 ****
**SRF (Yes:No)**				
IVA ^(3)^	10:04	7:07	6:08	0.29
IVBr ^(3)^	13:01	5:09	5:09	0.0021 **
IVF ^(3)^	10:04	5:09	4:10	0.051
**IRF (Yes:No)**				
IVA ^(3)^	3:11	0:14	0:14	0.040 *
IVBr ^(3)^	2:12	0:14	1:13	0.34
IVF ^(3)^	3:11	2:12	2:12	0.84

BCVA: best-corrected visual acuity, IOP: intraocular pressure, CSFT: central subfield foveal thickness, SRF: subretinal fluid, IRF: intraretinal fluid, ^(1)^ Wilcoxon signed-rank test, ^(2)^ Friedman test with a post hoc Dunn’s test, ^(3)^ chi-squared test; * *p* < 0.05, ** *p* < 0.01, **** *p* < 0.0001, ***** *p* < 0.00001.

## Data Availability

Researchers can contact Hisashi Matsubara (hmatsu@med.mie-u.ac.jp) for details of the protocol and results.
